# Real-time cortical dynamics during motor inhibition

**DOI:** 10.1038/s41598-024-57602-0

**Published:** 2024-04-03

**Authors:** Elias Paolo Casula, Valentina Pezzopane, Andrea Roncaioli, Luca Battaglini, Raffaella Rumiati, John Rothwell, Lorenzo Rocchi, Giacomo Koch

**Affiliations:** 1 Department of Clinical and Movement Neurosciences, University College London, London, WC1N 3BG UK; 2 Department of System Medicine, University of Tor Vergata, 00133 Rome, Italy; 3 Department of Behavioural and Clinical Neurology, Santa Lucia Foundation, 00179 Rome, Italy; 4 Department of Neuroscience and Rehabilitation, University of Ferrara, 44121 Ferrara, Italy; 5 Department of General Psychology, University of Padua, 35131 Padua, Italy; 6 Department of Medical Sciences and Public Health, University of Cagliari, 09124 Cagliari, Italy

**Keywords:** Cognitive neuroscience, Neurophysiology

## Abstract

The inhibition of action is a fundamental executive mechanism of human behaviour that involve a complex neural network. In spite of the progresses made so far, many questions regarding the brain dynamics occurring during action inhibition are still unsolved. Here, we used a novel approach optimized to investigate real-time effective brain dynamics, which combines transcranial magnetic stimulation (TMS) with simultaneous electroencephalographic (EEG) recordings. 22 healthy volunteers performed a motor Go/NoGo task during TMS of the hand-hotspot of the primary motor cortex (M1) and whole-scalp EEG recordings. We reconstructed source-based real-time spatiotemporal dynamics of cortical activity and cortico-cortical connectivity throughout the task. Our results showed a task-dependent bi-directional change in theta/gamma supplementary motor cortex (SMA) and M1 connectivity that, when participants were instructed to inhibit their response, resulted in an increase of a specific TMS-evoked EEG potential (N100), likely due to a GABA-mediated inhibition. Interestingly, these changes were linearly related to reaction times, when participants were asked to produce a motor response. In addition, TMS perturbation revealed a task-dependent long-lasting modulation of SMA–M1 natural frequencies, i.e. alpha/beta activity. Some of these results are shared by animal models and shed new light on the physiological mechanisms of motor inhibition in humans.

## Introduction

The inhibition of action is a fundamental executive mechanism of human behavior. The study of the neural basis underlying this process attracted great interest in cognitive neuroscience, leading to the definition of a neural network activated during the inhibitory processes. Neuroanatomical studies in animals and humans demonstrated a complex dynamic between the frontal cortex and the basal ganglia during the inhibition of a behavioral outcome^1^. In particular, during the execution of a Go/NoGo task, a prominent activation of the supplementary motor area (SMA) is observable^2^. Throughout the years, the critical involvement of this area during action inhibition has been confirmed with different Go/NoGo paradigms and using different techniques, such as event-related potentials (ERP)^3^, magnetic resonance imaging (MRI)^4^ and transcranial magnetic stimulation (TMS)^2^. In this scenario, the role of the SMA is of particular relevance, since it exerts proactive control in motor actions by regulating the level of excitability in the primary motor area (M1)^5,6^. The key role of SMA in controlling M1 output received direct demonstration both from animal models showing an increase of SMA local field power (LFP) during movement cancellation and reduced M1 excitability^5^, and from another study by Scangos and Stuphorn showing the influence of single SMA neurons over movement inhibition^7^.

In spite of the progresses reached so far, many questions regarding the “brain dynamics” related to action inhibition, i.e. the cascade of physiological events and their spatiotemporal distribution, are still unsolved. This lack of knowledge is mainly due to the technical limitations of the neurophysiological approaches used so far. For instance, although MRI provides a detailed description of the spatial origin of metabolic activity, it suffers from a poor temporal resolution (i.e., > 1 s)^8^. This is a critical problem given the highly dynamic nature of action inhibition processes that requires fast physiological changes in the brain. Recent advancements in the analysis of electroencephalography (EEG) provided a potentially powerful method to overcome this issue. However, spontaneous EEG might be not fully suited to draw causal conclusions about brain activity of discrete cortical areas, due its correlational nature and because it is susceptible to a number of artefacts that can produce unstable responses in the spatial or frequency domain^9^. These issues can be partially circumvented by the use of TMS, which allows functional perturbation of selected cortical areas^10,11^. So far, this technique has been mainly used in combination with electromyography (EMG) to investigate changes in corticospinal excitability associated with action preparation and inhibition, by measuring changes in motor evoked potentials (MEPs)^12,13^. The main problem with this approach is that MEPs cannot give direct information on associative motor and non-motor areas that are critically involved during action inhibition. In addition, it is important to consider that MEPs reflect the excitability of the whole corticospinal tract, including the spinal cord; thus, they cannot be considered an exclusive index of cortical excitability^14^.

In the present study we sought to overcome these limitations by combining TMS with EEG, a novel approach optimized to investigate brain dynamics in an effective and causative way^15^. By combining TMS of a specific brain area with simultaneous whole-scalp EEG recording it is possible to reveal the spatiotemporal dynamics of cortical activation of the stimulated and interconnected areas^16^. In the present study, we used the TMS–EEG approach to investigate the fast spatiotemporal dynamics occurring during motor inhibition. We chose to test a sample of older adults so that, in future studies, this sample can be comparable to pathological populations of the same age. We stimulated the primary motor area (M1), since this area M1 represents a final common stage of the complex activation of the motor network during inhibition; thus, insights obtained by its stimulation are critical in understanding the result of the cascade of events within the motor network during complex processes, such as inhibition^5,17,18^. During M1 stimulation, we recorded EEG dynamics occurring throughout the scalp while the participant performed a Go/NoGo task, with the aim to investigate TMS–EEG markers of motor inhibition. In addition, during the task, we characterized SMA–M1 connectivity dynamics at different timings of task execution: at rest, during the elaboration of the Go/NoGo cue, right after M1 stimulation and during movement execution/inhibition.

## Results

No participants reported any adverse effects due to the stimulation. The mean resting motor threshold (RMT) was 50.22 ± 4.94% of the maximum stimulator output; the mean induced e-field was 97.26 ± 3.3 V/m, ranging from a minimum of 92.3 and a maximum e-field of 102.8, well above the minimum threshold of 40 V/m we established a priori^19–21^.

### Behavioural performance

**Figure 1:**
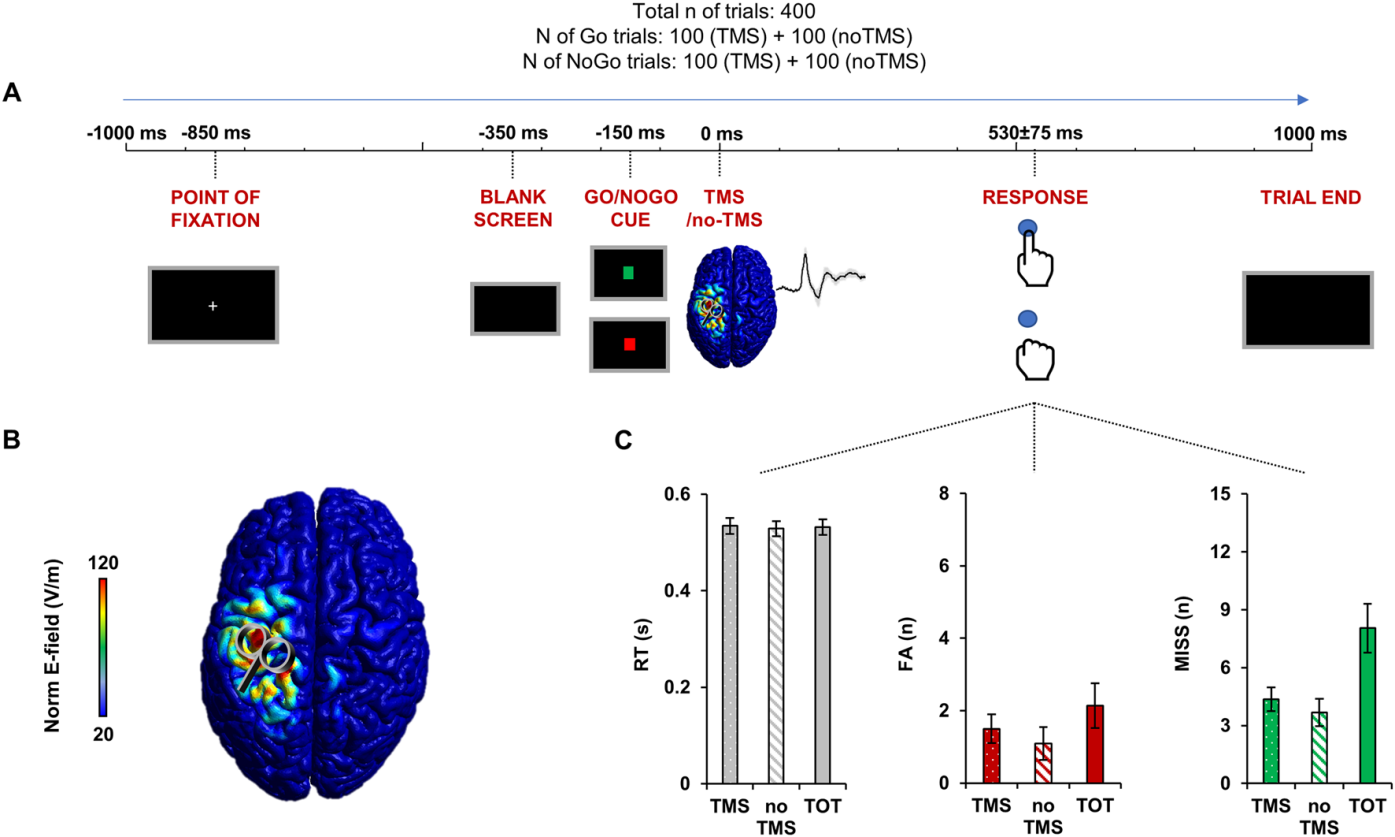
Go/NoGo task and TMS–EEG setup. Panel (**A**): schematic representation of a Go/NoGo trial during TMS–EEG. Each trial started with a point of fixation (− 850 ms in respect to TMS) followed by a blank screen (− 350 ms in respect to TMS) and by a cue (− 150 ms in respect to TMS) that could depict a Go trial (green square) or a NoGo trial (red square). The cue lasted 150 ms, right after there was a TMS pulse delivered over the left primary motor area (M1, 0 ms). After 530 ± 75 ms (mean ± SD) there was the task response (button press in a keyboard) in the Go condition. Panel (**B**): estimated electric field induced in the cortex during TMS of M1. Panel (**C**): averaged behavioural performance of the 22 participants at the Go/NoGo task during TMS trials, non-TMS trials and total (TOT: TMS + non-TMS) in terms of mean reaction time (RT), false alarms (FA) and missed responses (MISS). Error bars depict SEM.

Figure 1C depicts the behavioural performance at the Go/NoGo task in terms of reaction times (RT), false alarms (FA) and missed responses (MISS). We first assess if TMS influenced the RT duration, the number of FA and MISS by comparing the trials with TMS versus without TMS. Paired t-tests did not reveal any influence of TMS in the RT duration (TMS: 534 ± 77 ms; no TMS: 528 ± 73 ms; *p* = 0.143); % of FA responses (TMS: 0.53 ± 0.14%; no TMS: 0.38 ± 0.16%; *p* = 0.119) and % of MISS responses (TMS: 1.55 ± 0.22%; no TMS: 1.31 ± 0.25%; *p* = 0.122). The general behavioural performance was: 531 ± 75 ms for RT; 0.76 ± 0.22% of FA responses and 2.87 ± 0.45% of MISS responses.

### SMA–M1 connectivity

**Figure 2:**
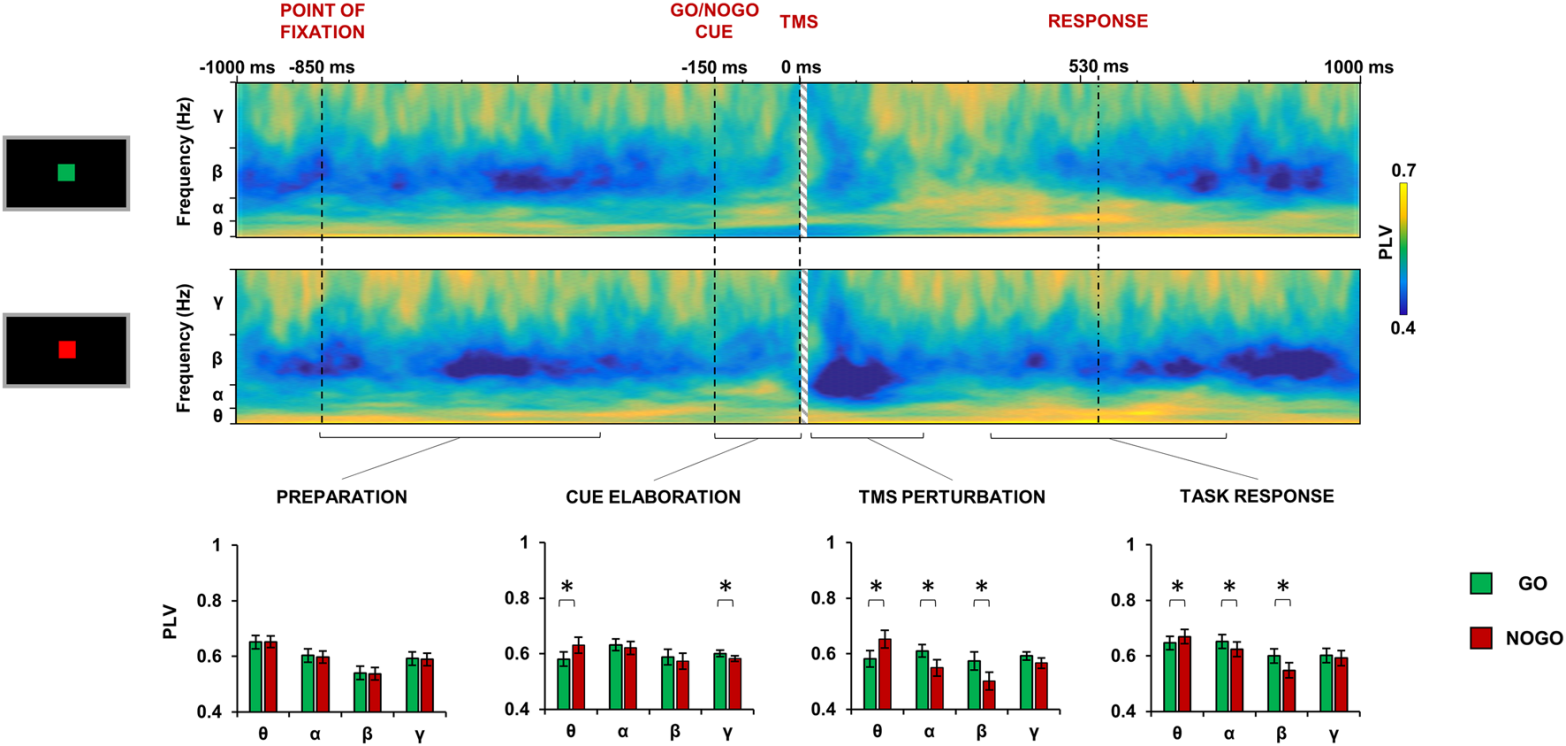
Phase-locking value (PLV) computed between the left primary motor cortex and supplementary motor area (SMA) throughout the Go/NoGo task in the Go (upper panel) and NoGo condition (lower panel). Bar charts depict the mean PLV in the four task phases (preparation, TMS perturbation, cue elaboration, task response) for the Go (green bars) and NoGo condition (red bars) in the four frequencies of oscillation (theta, alpha beta, gamma). Error bars depict SEM. *p < 0.05.

Figure 2 depicts the phase-locking value (PLV) dynamics reflecting SMA–M1 connectivity during the TMS trials, in the Go and NoGo condition. The ANOVA aimed at investigating overall differences in oscillatory synchronization among the four phases of the task (preparation, cue elaboration, TMS perturbation and movement response) in the Go and NoGo condition, revealed a significant task × condition × frequency interaction [F(9,189) = 5.526; *p* < 0.001; η^2^ = 0.208] (Fig. 2). Post-hoc analysis showed no difference during the “preparation” phase (all post-hoc ps > 0.05); during the “cue elaboration” phase, in the NoGo condition, we observed an enhancement of theta-PLV (NoGo–Go mean difference: − 0.060 ± 0.023; post-hoc *p* = 0.017) and a lower level of gamma-PLV (NoGo–Go mean difference: − 0.018 ± 0.007; post-hoc *p* = 0.017), in respect to the Go condition. During the “TMS perturbation” phase, in the NoGo condition, the enhancement of theta-PLV was still significant (NoGo–Go mean difference: − 0.071 ± 0.026; post-hoc *p* = 0.013), whereas alpha-PLV (NoGo–Go mean difference: − 0.061 ± 0.018; post-hoc *p* = 0.003) and beta-PLV (NoGo–Go mean difference: − 0.073 ± 0.019; post-hoc *p* = 0.001) were reduced, compared to the Go condition. These effects were still significant during the “task response” phase, with a higher theta-PLV (NoGo–Go mean difference: 0.023 ± 0.011; post-hoc *p* = 0.046) and lower alpha-PLV (NoGo–Go mean difference: − 0.028 ± 0.013; post-hoc *p* = 0.041) and beta-PLV (NoGo–Go mean difference: − 0.052 ± 0.013; post-hoc *p* = 0.001) in the NoGo condition, in respect to the Go condition.

### M1 TMS-evoked potentials

**Figure 3:**
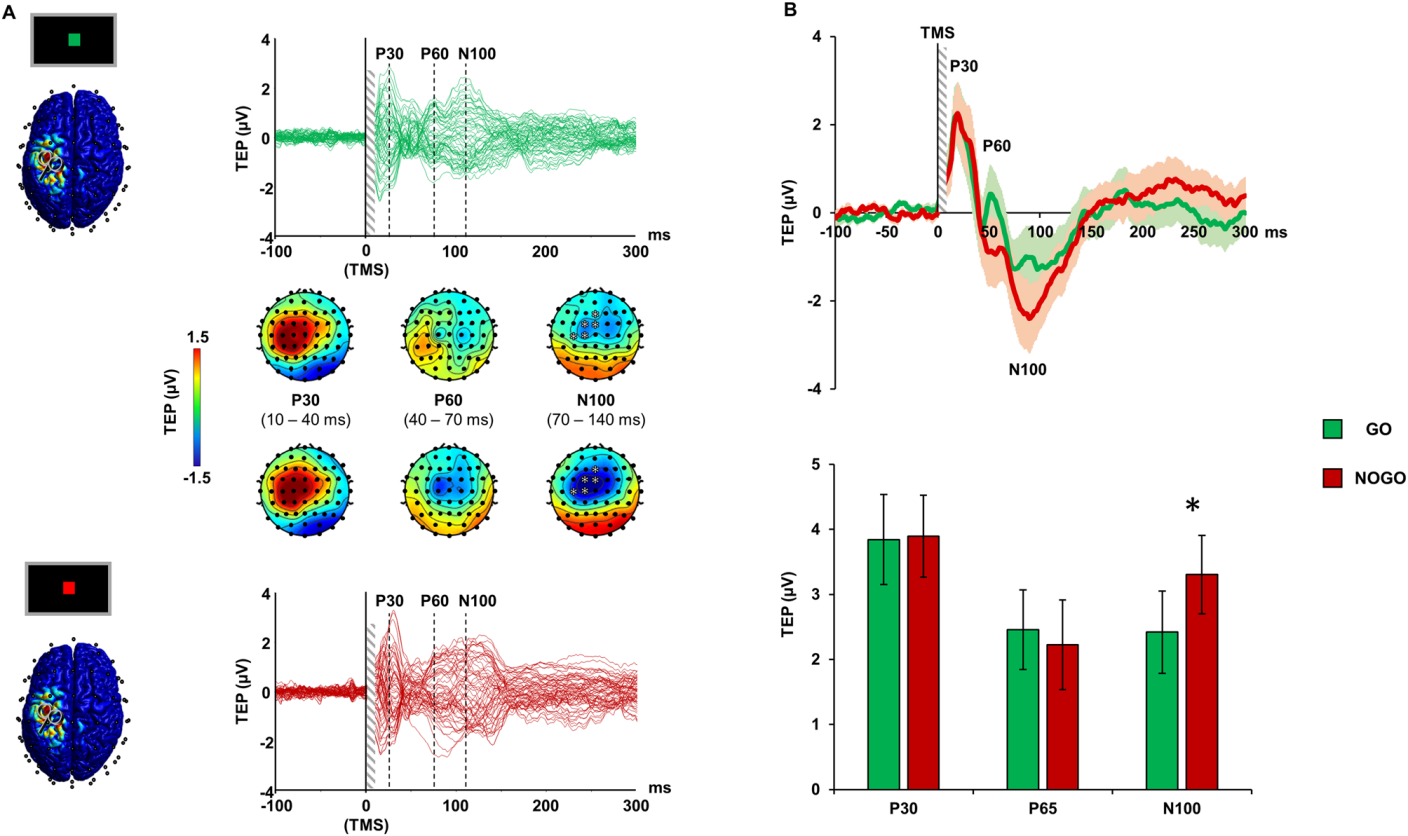
TMS-evoked potentials (TEPs) during Go/NoGo task. Panel (**A**): butterfly plots of the TEPs recorded during Go (green lines, upper plot) and NoGo condition (red lines, below plot) with the relative scalp voltage maps distribution. Panel (**B**): the upper chart depicts the TEP recorded over a significant cluster of electrodes indicated in panel A during Go (green line) and NoGo condition (red line) shaded lines depict SEM. The below bar chart depict the TEP peak amplitude (expressed in absolute value) during Go (green bars) and NoGo condition (red bars). Error bars depict SEM. *p < 0.05.

Figure 3 depicts the TEPs evoked during the Go/NoGo task over all the scalp (panel A) and locally to the stimulated M1 (panel B). TEPs consisted of three main components peaking around 30 (P30), 60 (P60) and 100 (N100) ms after TMS. These three components follow a well-known spatio/temporal pattern reported in previous works^14,16,22^. Specifically, the first component consisted in a prominent positivity peaking around 30 ms after TMS, focused on the site of stimulation; this was followed by a dipole centred over the site of stimulation characterized by a contralateral negativity and by a positivity centred in the stimulated hemisphere at around 60–65 ms after TMS. Around 100 ms after TMS, a prominent negativity is observable in a large cluster of left fronto-central electrodes; this is a highly-reproducible component usually termed as N100. Global analysis of TEPs revealed that only the latter component was modulated by the Go/NoGo task. Specifically, the TMS-evoked N100 was larger during the NoGo condition in a cluster of five fronto-central electrodes, i.e., Fz, FCz, FC1, C1 and C3 (Fig. 3A). Analysis of the same components locally to the cluster showed a significant TEP peak × condition interaction [F(1.664, 34.935) = 4.896; *p* = 0.018; η^2^ = 0.189]. Post-hoc analysis showed that the N100 component was significantly higher during NoGo trials compared to Go trials (mean difference: 0.884 ± 0.359 µV; post-hoc *p* = 0.022) whereas no modulation was observable for P30 and P60 TEPs (all ps > 0.05; Fig. 3B).

**Figure 4:**
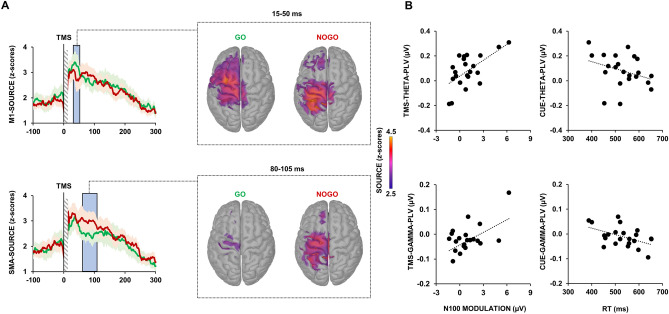
Source reconstruction of cortical activity and linear correlations. Panel (**A**): left charts depict source reconstruction of cortical activity in the time domain during Go (green lines) and NoGo condition (red lines), shaded lines depict SEM. Light blue squares indicate the significant differences between the Go and NoGo condition. Central brain maps depict the spatial reconstruction of the significant differences. Panel (**B**): linear relationships between SMA–M1 PLV, TMS-evoked N100 modulation and RT.

Figure 4A depicts source analysis locally to M1 and SMA ROIs (see “Methods” section) and at global level. Significant changes of source activity were observable within an earlier time windows, between 15 and 50 ms, for M1, and within a later time window, between 80 and 105 ms, for SMA. Specifically, we found an increase in source activity over M1 (from 39 to 50 ms after TMS; mean t(20) = − 1.846; mean *p* = 0.039) during the Go condition, compared to the NoGo condition; whereas an increase of source activity was visible over the SMA during the NoGo condition, compared to the Go condition (from 85 to 105 ms; mean t(20) =  − 1.812; mean *p* = 0.042).

### Behavioural/Neurophysiological linear relationships

Figure 4B depicts the linear relationships between the modulation of TMS-evoked N100, the modulation of SMA–M1 PLV and the RT. The regression analysis showed that the amplitude of the TMS-evoked N100 was predicted by theta and gamma SMA-M1 PLV during TMS perturbation [F(1,20) = 12.842; *p* < 0.001]. Specifically, predictors analysis showed that both theta and gamma PLV were positively related to the modulation of TMS-evoked N100 (theta-PLV: β = 8.957; t = 4.136; *p* = 0.001; gamma-PLV: β = 11.304; t = 2.932; *p* = 0.009). Correlation analysis revealed that the individual RT was inversely correlated to the modulation of the SMA–M1 PLV in the gamma (r = − 0.5, *p* = 0.009) and in the theta band (r = 0.423, *p* = 0.025) during cue elaboration.

### Event-related potentials

**Figure 5:**
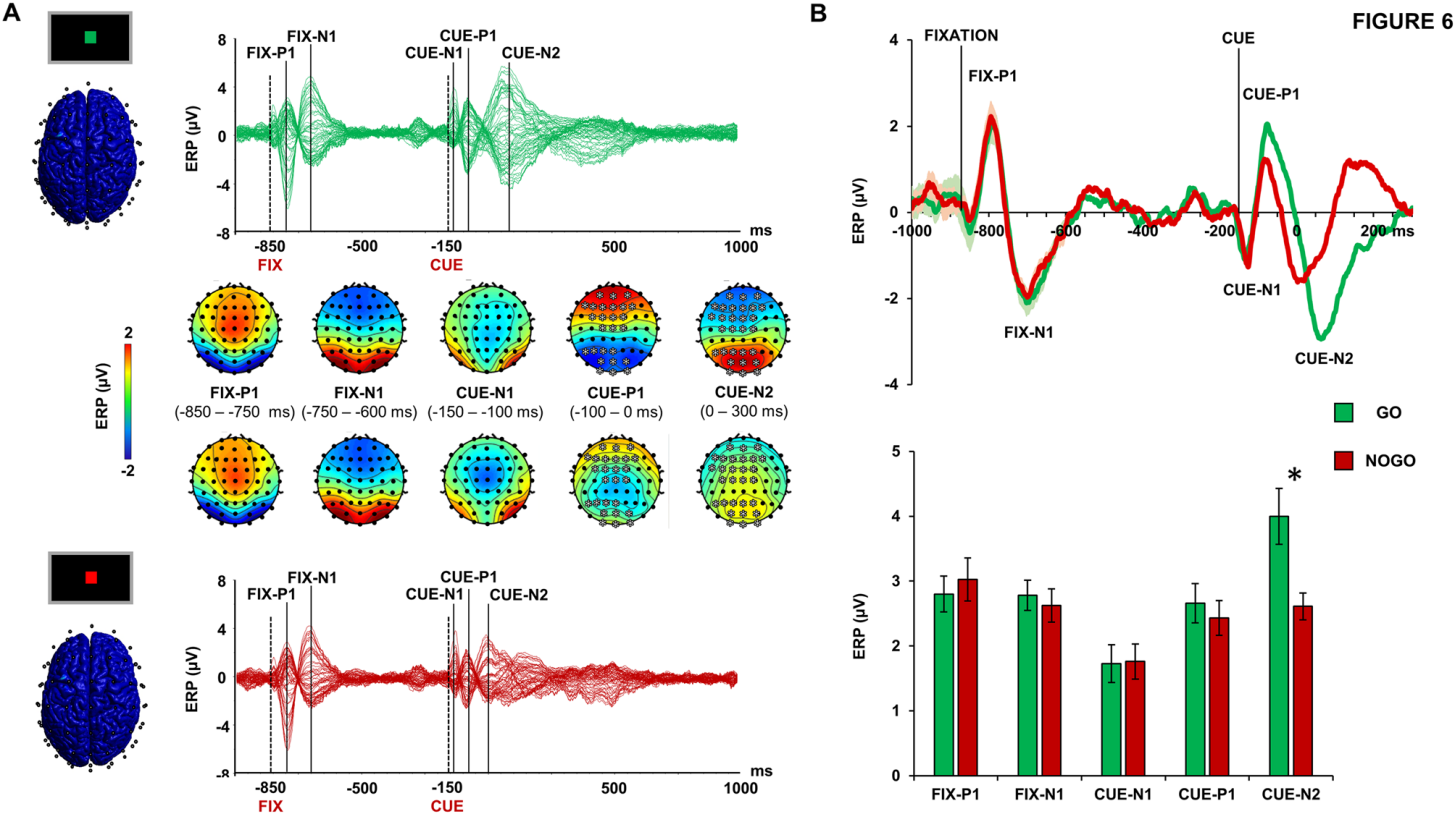
Event-related potentials (ERPs) during Go/NoGo task. Panel (**A**): butterfly plots of the ERPs recorded during Go (green lines, upper plot) and NoGo condition (red lines, below plot) with the relative scalp voltage maps distribution. Panel (**B**): the upper chart depicts the ERP recorded over a significant cluster of electrodes indicated in panel A during Go (green line) and NoGo condition (red line) shaded lines depict SEM. The below bar chart depict the ERP peak amplitude during Go (green bars) and NoGo condition (red bars). Error bars depict SEM. *p < 0.05.

Figure 5 depicts the ERPs evoked during the Go/NoGo task over all the scalp (panel A) and locally to the medial frontal cortex (panel B). The ERP response was characterized by the presence of specific components related to the appearance of point of fixation (FIX-P1 and FIX-N1), which were clearly visible right after the appearance of the fixation cross (− 850 ms), and to the appearance of the cue (− 150 ms) showing a Go or NoGo condition (CUE-N1, CUE-P1, CUE-N2). All these components were mainly distributed in medial frontal electrodes as reported by previous study using a similar paradigm^3,23,24^. Global analysis of ERP showed that the CUE-P1 and the CUE-N2 components were modulated by the Go/NoGo task. Specifically, we observed a higher CUE-P1 and CUE-N2 amplitude during the Go condition in two large bilateral clusters of fronto-central (AFz, AF3, AF4, F5, F3, F1, Fz, F2, F4, FC3, FC1, FCz, FC2, C1, Cz, C2 for CUE-P1; AFz, AF3, AF4, F5, F3, F1, Fz, F2, F4, FC5, FC3, FC1, FCz, FC2, C3, C1, Cz, C2 for CUE-N2) and posterior electrodes (P5, P3, P1, Pz, PO1, POz, PO2, O1, Oz, O2 for CUE-P1; P5, P3, P1, Pz, P2, PO1, POz, PO2, O1, Oz, O2 for CUE-N2). Analysis of the same components at local fronto-central level, showed a significant ERP peak × condition interaction [F(2.009, 42.194) = 8.035; *p* = 0.001; η^2^ = 0.277]. Post-hoc analysis showed that CUE-N2 was significantly higher during the Go condition compared to the NoGo (mean difference: 1.386 ± 0.391 µV; post-hoc *p* = 0.002; Fig. 5B).

## Discussion

In this study we used the combined TMS–EEG approach to reconstruct the brain dynamics occurring during the performance of a Go/NoGo task. We first evaluated the real-time connectivity between the SMA and the M1 throughout the different phases of the task, i.e., preparation, cue elaboration, TMS perturbation, task response. There was no difference between conditions in the preparation phase, when the participant was still unaware whether a Go or NoGo cue would be presented. During cue elaboration, we observed an increase of theta-frequency connectivity in the NoGo condition, in respect to the Go, while the opposite was true for gamma-frequency connectivity. Frontal theta activity has been often related to attentional process^25^, conflict monitoring^26^ and cognitive control^27^. These processes are involved in the Go/NoGo task and, in particular, are critical for successful response inhibition. Accordingly, previous studies using standard EEG recordings during Go/NoGo tasks frequently reported an increased medial frontal theta activity during NoGo trials^28,29^ and a modulation of specific event-related potentials, namely the NoGo-P1 and the NoGo-N2^30^. Both these electrophysiological signatures were present in our results, confirming the reliability of the Go/NoGo paradigm we adopted, and they likely reflect the same cognitive process in the time-domain, i.e. P1/N2, and in the frequency-domain, i.e., mid-frontal theta activity^28^. As regards the functional role of the P1/N2 complex, previous studies suggested that this potential could reflect an activation of the sensorimotor areas prior to the motor execution^31^. Accordingly, previous ERP studies using a Go/NoGo task, observed an amplitude attenuation of the P1/N2 complex^31^, as well as a reduction in its duration^32^, during NoGo trials, confirming the supposed role of this potential in motor preparation. Notably, both of these findings are clearly observable in our results. As regards the role of theta activity in motor inhibition, this has been confirmed in several EEG studies using both spectral measures, in terms of power^33^ and phase^34^, as well as in connectivity investigations^35,36^. For instance, Prochnow and colleagues, found an increase of connectivity between fronto-medial areas in the theta band during response inhibition (Prochnow et al., 2022). Along the same lines, Smit and colleagues, in their study found an increase of functional theta connectivity related to the execution of tasks involving conflict monitoring and response inhibition. On the other hand, the role of gamma activity is more debated. Our results showed that gamma-mediated connectivity was increased when participants were exposed to a Go cue. In animals, the relationship between gamma power and movement preparation has received support from studies using intracellular recordings^37,38^, suggesting a specific role of high-frequency gamma activity during motor inhibition/execution (for a review see Wardak 2011). Alternatively, it could be hypothesized that the observed changes in gamma activity may reflect a re-balance of the theta/gamma coupling in response to the changes in theta activity, given that these frequencies naturally occur in the same brain regions and interact with each other^40^. This latter hypothesis is supported by a previous study conducted with intracellular recordings from the SMA of monkeys while performing a motor task^5^. Here, the authors observed a re-balance of low- and high-frequency power recorded from the SMA just before that the execution of a movement, with the latter being increased during movement preparation, whereas slow frequencies were reduced^5^. Interestingly, the individual levels of low- and high-frequency power recorded from the SMA correlated with the timing of the monkeys’ responses, a result that is in line with what we observed in our correlation analysis. Here, we found that the modulation of both theta- and gamma-frequency connectivity was inversely related to the timing of the task response, i.e., higher modulation of theta- and gamma-PLV were associated to faster motor responses. We also found that the TMS-evoked N100 amplitude modulation was linearly dependent from the individual level of theta- and gamma-frequency PLV. Although the nature of this potential has not been fully elucidated, previous studies suggested that the TMS-evoked N100 origins from the slow GABA_B_-mediated inhibitory post-synaptic potentials following the stimulation of M1, as revealed by pharmacological studies using GABA_B_ receptor agonists^41,42^ or investigating several pathological conditions affecting cortical inhibition^22^. In light of this background, we could speculate that the neuronal depolarisation generated by TMS involved mostly inhibitory GABAergic neurons that were more excitable during the “NoGo” condition. The activation of interneurons in the SMA releases GABA across the numerous synapses to the M1, and this induces inhibition in the form of post-synaptic potentials, which are reflected in the larger TMS-evoked N100 amplitude in NoGo condition, a result in line with previous studies using a similar approach^43,44^. Accordingly, the spatial reconstruction of this potential showed that it was prominent over the stimulated motor area and, in particular, over the SMA, as revealed by the source analysis. Notably, the occurrence of such activity was approximately 200–250 ms after the visual cue and about 400 ms before the average RT in the Go condition. Thus, from a cognitive perspective, this activity likely reflect an early motor preparation, during which the SMA play a key role^45^. The local source analysis over M1 revealed also an early higher activation (20–50 ms after TMS) during the Go condition, compared to the NoGo condition. TMS-evoked activity in this early time window is thought to reflect local excitability of the stimulated area^42,46^; therefore, when participants elaborate the Go cue and prepare to move, the excitability of M1 increases. Notably, this activation is not present in the SMA; this result suggests a dissociation in the role of M1 and SMA, the first being activated only during preparation to move and the latter showing prominent activation only during movement inhibition.

Another result that we observed was the task condition-dependent modulation of alpha- and beta-frequency connectivity during the “TMS perturbation” and the “task response” phases of the task. The “TMS perturbation” phase comprised the first 150 ms after the TMS pulse (excluding the first 10 ms, which were interpolated). This time window was chosen based on the large TMS–EEG literature showing that TMS produces a time/frequency response on the EEG signal lasting about 150 ms^16^. It is important to note that, in our study, TMS was used as a probe to assess cortical activity and did not interfere with the task performance. Indeed, in our study TMS was delivered at a subthreshold intensity and it did not evoke any MEP, as revealed by the concomitant EMG recordings during the task. Accordingly, we did not observe any difference in the performance of the task during TMS or non-TMS trials. But what is the effect of TMS on the oscillatory activity? It is thought that TMS acts like a “magnifier” on the spontaneous EEG signal that boosts the naturally occurring frequencies in a specific brain area, without corrupting the natural pattern of response^19,47^, a notion confirmed by our results. Indeed, the frequency pattern observed during the “TMS perturbation” was the same as that of the immediately preceding “cue elaboration” phase, with the only difference that the TMS pulse boosted the power of the oscillatory synchronization, revealing some differences that were less evident in the preceding phase. Specifically, in addition to the observed modulation of theta- and gamma-connectivity, we observed that alpha- and beta-connectivity were respectively reduced or increased depending on the occurrence of NoGo or Go condition. The importance of alpha- and beta- oscillations in motor areas has been already highlighted by previous studies investigating natural frequencies over motor regions^14,22,48–51^. Using TMS as a probe, these studies revealed that the perturbation of M1 and associative motor areas resulted in a main synchronized oscillatory activity in the alpha/beta range, i.e., the so-called natural frequency^47^. In this context, it seems that once the participant is preparing his/her motor response, there is an increase of SMA–M1 connectivity in the natural frequencies of M1, likely resulting from the increase in the excitability of this area. Such a pattern is still present throughout the entire time window of movement preparation until the execution of the motor response. On the other hand, when the participant has to inhibit his/her response, we observed a reduction of the movement-related alpha/beta-connectivity.

Our study presents some limitations. First, we chose to test a sample of middle age healthy individuals, this potentially bias our behavioural results, as we observed a slightly longer average RT, compared to studies in young healthy volunteers^52,53^. Second, we did not have individualized MRIs of our participants; therefore, it was not possible to localize the SMA based on individual anatomy. To minimize errors due to individual variabilities, we localized the SMA-ROI based on the individual M1 hotspot, which was functionally targeted with MEPs. Then, we further verified the correspondence with a well-known brain atlas^54^ to define the SMA-ROI for source analysis. Our localization was also verified comparing a recent study in which we specifically optimized the targeting and stimulation of the SMA using TMS–EEG^55^. Another limitation lies in the relatively low spatial resolution of our EEG recording, which did not allow to discriminate specific portions of the SMA. Indeed, the localization of our source analysis based on the minimum norm estimate method, with signal-to-noise ratio of 3, was about 1.8 cm^56,57^. Moreover, we focused on the role of the SMA, since this area seems to play the prominent role in motor inhibition and received great attention in cognitive neuroscience. However, several other nodes of the motor network, such as the inferior frontal gyrus and the premotor cortex, are known to be critically involved in motor inhibition. Future studies need to better characterize the role of these nodes in motor inhibition and control. Finally, another potential source of bias could arise from the contamination of TMS–EEG signals by auditory evoked potentials (AEP)^58,59^. In this regard, we used a masking noise, ear defenders and a 0.5 cm foam layer underneath the coil, a setup shown to suppress AEPs when TMS is applied at an intensity similar to the one adopted in the present study. In conclusion, this study aimed to reconstruct the spatiotemporal dynamics occurring in M1 and SMA during planning, execution and inhibition of voluntary movement. We observed specific TMS–EEG signatures that were modulated during these processes, i.e., SMA–M1 connectivity and TMS-evoked N100 amplitude. Some of these results are shared by animal models and shed new light on the underlying physiological mechanisms of motor inhibition in humans.

## Materials and methods

### Participants

Twenty-two healthy volunteers with age between 45 and 70 years (10 females; mean age ± SD, 58.5 ± 11 y) were enrolled in the study. They were all right-handed^60^, with normal visual acuity, and were naive to the purpose of the experiment. All subjects gave their written informed consent before the experiment and did not have exclusion criteria for TMS^61^. The experimental protocol was approved by the ethics committee of the Fondazione Santa Lucia and was carried out in accordance with the ethical standards of the 2013 Declaration of Helsinki. The appropriateness of our sample size was established by a power calculation performed with G*Power software, from the effect size reported by a previous study conducted using a similar paradigm^43^. In this study, an effect size of 0.37 (obtained as the post–pre means over pooled standard deviation) was observed for the Go/NoGo task effect in the TMS-evoked N100 amplitude, testing 6 participants. Thus, given that our sample was of 22 participants, it was plausible to estimate an effect size of at least 0.6, with type I error alpha = 0.05 and power of 0.9.

### General procedure

All the participants underwent an experimental session lasting about 90 min. Participants were asked to perform a computerized Go/NoGo task in which they were instructed to perform a motor response with their right dominant hand, i.e., pressing a button on a response box as fast as they could (Go condition), or to withhold it (NoGo condition) based on a visual cue displayed on a computer screen (see Go/NoGo task paragraph for technical details) (Fig. 1). Participants sat in a comfortable armchair at a distance of 80 cm from the computer screen. During the task, EEG activity was recorded from the whole scalp (see TMS and EEG paragraphs for technical details). To avoid auditory responses caused by TMS, participants were given earphones that continuously played a masking noise composed of white noise mixed with specific time-varying frequencies of the TMS click^59,62^. The masking noise volume was adjusted to ensure that participants could not detect the TMS click, or as much as tolerated (always below 90 dB). In order to further reduce the impact of the TMS click on EEG signal, we placed ear defenders (SNR = 30) on top of the earphones; this approach has been proven to be effective in suppressing the auditory-evoked potential that is caused by the TMS click^59^. To avoid bone conduction of the TMS click and to reduce the sensation caused by coil vibration, we placed a 0.5-cm foam layer beneath the coil^59,63^.

### Go/NoGo task

We used a standard computerized Go/NoGo task with visual stimuli programmed with Psychtoolbox running in a MATLAB environment (Version 2017, MathWorks Inc., Natick, USA) (Fig. 1A). Each trial of the task started with a point of fixation, i.e. a white cross of 3 × 3 cm displayed at the center of a screen for 500 ms, followed by a blank screen lasting 200 ms, and by a cue, i.e., a square of 3 × 3 cm displayed at the centre for 100 ms. The cue could be a green square, indicating a Go trial, or a red square indicating a NoGo trial. Participants were instructed to press the spacebar with their right index finger on the computer keyboard as fast as possible, as soon as they saw the green square, or to withhold their response in case the red square was projected. The inter-trial interval was randomized between 2 and 4 s. The task was composed of 400 trials, 50% of which (200) were Go and 50% (200) were NoGo trials. Trials were further subdivided in TMS trials, where a TMS pulse was delivered over the left M1 150 ms after the cue, and trials where no TMS was given. The interval of 150 ms between the visual cue and the TMS pulse was chosen based on previous works using a similar paradigm^6,64^ and on the well-established notion on which the elaboration of a visual stimuli, even when complex, is produced within about 150 ms after the stimulus onset, as revealed in humans (for a review see^65^) and in animal studies^66^. Thus, in total, there were 400 trials divided as follows: 100 TMS-Go trials, 100 TMS-NoGo trials, 100 Go trials, 100 NoGo trials. To avoid fatigue and keep concentration at an optimal level, the whole set of trials was divided in two runs of approximately 8 min each, separated by a break of 2 min. For each trial, a number of behavioral variables were recorded, including reaction times (RT), false alarms (FA, i.e., Go responses in NoGo trials), and missed responses (MISS, i.e., missed responses in Go trials).

### Transcranial magnetic stimulation

TMS was carried out with a Magstim Rapid^2^ device connected to a 70 mm figure-of-eight coil (Magstim Company Limited, Whitland, UK), which produces biphasic pulses of ∼320 µs duration. For M1 stimulation, the coil was positioned tangentially to the scalp at an angle of 45° from the midline, with the handle pointing backward, over the hand motor area of the left M1, defined as the point where stimulation evoked the largest MEPs of the right first dorsal interosseous (FDI) muscle. Stimulus intensity was set to 90% of RMT, which was defined as the intensity able to yield an MEP of at least 50 µV peak-to-peak amplitude in 5 out of 10 consecutive trials. To ensure that this intensity was sufficient to evoke a reliable cortical response, i.e. > 40 V/m^19,20,47^, we computed the induced E-field with SimNIBS software^67^ using an MNI standard brain (ERNIE) as an anatomical reference^68^ (Fig. 1B). To ensure a high degree of reproducibility across neurophysiological assessments, the coil position was constantly monitored throughout the stimulation by using a Brainsight neuronavigation system (Rogue Research Inc, Montreal, Canada) coupled with a Polaris Spectra optical measurement system (Northern Digital Inc, Waterloo, Canada).

### Electroencephalographic recordings and pre-processing

EEG was recorded using a TMS-compatible DC amplifier (BrainAmp MR plus, BrainProducts GmbH, Munich, Germany). The amplifier was optically connected to a PC with BrainVision Recorder, through which the EEG was monitored online, and to a 64-channels EEG cap (EasyCap Inc., Herrsching, Germany). The EEG was continuously recorded with 62 TMS-compatible Ag/AgCl passive pellet electrodes mounted on the cap according to the 10–20 international system. Recordings were on-line referenced to the tip of the nose and the ground electrode was placed on FPz. Skin impedance was kept below 5 kΩ, the sampling frequency was 5000 Hz.

TMS–EEG and EEG signals (the latter for epochs were TMS was not applied) were pre-processed with the same procedures except that, for the TMS–EEG signals, we first removed the signal between − 1 and + 10 ms from the pulse and applied a cubic interpolation on the missing segment. The continuous EEG signal was then band-pass filtered between 1 and 80 Hz (Butterworth zero phase filter). A 50 Hz notch filter was also applied to reduce electrical line noise. After this, the continuous EEG signal was downsampled to 1000 Hz and segmented into epochs starting 1000 ms before the TMS pulse and ending 1000 ms after it. Then, epochs were demeaned (by subtracting the average signal amplitude to each time point), visually inspected, and those with excessive noise were excluded from the analysis (less than 5% for each participant). Independent component analysis (FastICA) was applied to the segmented EEG signal to identify components reflecting continuous muscle activity, eye movements, blink-related activity, and residual TMS-related artefacts (due to activation of craniofacial muscles and voltage decay). Artefact-related components were identified by their scalp distribution, frequency, timing and amplitude, and then removed following previously published indications^69,70^. After the pre-processing steps, each participant has a TMS–EEG dataset and an EEG dataset, of about 200 trials, recorded during the performance of the Go/NoGo task.

### Electroencephalographic analyses

TMS–EEG and EEG analyses were performed with EEGLAB v.21^71^ and Brainstorm toolbox^72^, running in a MATLAB environment (Version 2017b, MathWorks Inc., Natick, USA).

To assess SMA–M1 connectivity throughout the task execution, we computed the PLV between M1 and SMA. PLV is a commonly used measure of phase synchronization in oscillatory activity between two signals. If the phases of the two signals are strongly coupled, then the PLV will approach a value of 1, otherwise it will be close to zero^73^. For this analysis, we first performed a time/frequency decomposition of the entire TMS–EEG epoch (from − 1 to 1 s after TMS) based on a complex Morlet wavelet (cycles = 3; frequency resolution = 1 Hz from 4 to 50 Hz; temporal resolution = 1 ms). Then we computed the PLV in the theta (4–7 Hz), alpha (7–13 Hz), beta (13–30) and gamma frequency (30–50 Hz) for the entire time window from − 1 to 1 s after TMS. To assess connectivity changes during the critical phases of the task, we also averaged the PLV within four specific time windows (Fig. 1): during the “preparation” phase, i.e., from the appearance of the fixation point (− 850 ms) to its disappearance (− 350 ms); during the “cue elaboration” phase, i.e., from the appearance of the cue (− 150 ms) to the TMS pulse (− 1 ms); during the “TMS perturbation” phase, i.e., from the TMS pulse (10 ms, considering that from − 1 to 10 ms the signal was removed and interpolated, see EEG pre-processing paragraph) to the end of the TMS-evoked response (150 ms, see “Results” section); during the “task response” phase, i.e., from 150 to 650 ms. This last time window was chosen based on the average reaction time and the relative standard deviation (531 ± 75 ms, see “Results” section). Signals were estimated in the source space by the two anatomical regions of interest. We chose this method since the PLV may be biased by volume conduction effects, which can be avoided by calculating source-space connectivity with the minimum norm estimate method^74,75^. To estimate the spatial source of cortical activity, we fitted a distributed source model consisting of 15,000 elementary current dipoles. These dipole sources were distributed at each vertex of a tessellated cortical mesh template surface derived from a standard 1 mm resolution brain (Colin27) in the MNI space, as provided by Brainstorm toolbox. First, the head model for source imaging was implemented according to the OpenMEEG boundary element method^76^. Based on this head model, the inverse problem was solved using current density maps. Noise covariance for source reconstruction was obtained separately for each subject from a baseline window ranging from − 850 to − 350 ms in respect to TMS. The estimated source distribution was averaged across all the participants and two regions of interest (ROIs), corresponding to M1 and SMA, were chosen a priori to analyse source activity. M1-ROI was established based on each stimulated hotspot of the 22 participants tested and was centred over the following MNI coordinates: x = − 31.3 ± 3.6; y = − 15.4 ± 2.2; z = 75.8 ± 3.4. The SMA-ROI MNI coordinates were selected according to the definition of the Desikan-Killiany atlas: x = − 10; y = − 2; z = 77^54^.

To assess cortical reactivity during the Go/NoGo task, we first averaged all the TMS–EEG epochs recorded during the task to obtain a cortical response comprising both TEPs and ERPs, which we termed TERPs. Then, to obtain a pure TEP response, we subtracted the averaged EEG responses recorded during the task, i.e., ERPs, from the TERP response. For TEP and ERP analyses we considered a time window of 2 s. Source distribution was also computed for TEP analysis in order to reconstruct cortical reactivity in the two ROIs of interest, i.e., M1 and SMA.

### Experimental design and statistical analysis

Parametrical analyses were performed using SPSS version 22 (SPSS Inc., Chicago, USA). Non-parametrical analyses were performed using EEGLAB^71^ and Brainstorm toolbox^72^ running in a MATLAB environment (version 2021, Mathworks inc., Natick, USA). Normal distributions of residuals were assessed by means of the Shapiro–Wilks' test, on which we based the use of parametrical or non-parametrical statistics. Sphericity of the data was tested with Mauchly's test. When sphericity was violated the Greenhouse–Geisser correction was used. Level of significance was set at α = 0.05.

We first assessed whether there was any change in the behavioural performance of the task related to the occurrence of the TMS pulse. To this aim, we used dependent t-tests or signed-rank Wilcoxon tests comparing the average RT, FA and MISS in the TMS–EEG trials and in the EEG trials.

To assess changes in SMA–M1 connectivity related to the occurrence of Go or NoGo task condition, we used PLV as a dependent variable. PLV was analysed with a 3-way repeated-measures ANOVA with “task phase” (preparation; cue elaboration; TMS perturbation; task response); “task condition” (Go, NoGo) and “frequency” (theta, alpha, beta, gamma) as within-subject factors. Post-hoc analysis were conducted with dependent t-test corrected with Sidak method.

To assess the changes in cortical activity related to the occurrence of Go or NoGo task condition, we used TEP amplitude as dependent variable. Analysis of cortical activity at global level, i.e., throughout the whole set of electrodes, was performed with a non-parametric, cluster-based permutation statistics conducted at specific time windows of interest (TOI) based on visual inspection of TEPs (see below), for each individual electrode. This method performs a non-parametric statistical test by calculating Monte Carlo estimates of the significance probabilities from two surrogate distributions constructed by randomly permuting the two original conditions data for 3000 times. The clusters for permutation analysis were defined as the two (or more) neighbouring electrodes in which the statistics value at a given TOI exceeded a threshold of *p* = 0.05^77^. This analysis was conducted by comparing TEP amplitude within their TOIs (i.e., 10–40 ms for P30; 40–70 ms for P60; 70–130 ms for N100; see results section) in the two task conditions (Go and NoGo). Analysis of activity at the local level was conducted within a cluster of left fronto-central electrodes resulting significant from cluster-based analysis (see “Results”) and was performed with a 2-way repeated-measures ANOVA with “task condition” (Go trials vs NoGo trials) and “TEP peak”, computed as the maximum absolute value reached by the three components (P30, P60, N100; see “Results”) as within-subject factors. Post-hoc analysis were conducted with dependent t-test corrected with Sidak method.

To assess real-time changes of cortical activity at source level, we conducted a time point-by-time point comparison within the same time window of TEPs, i.e., from − 100 to 300 ms after TMS, over M1 and SMA ROIs. This analysis was conducted using multiple dependent t-tests. Given the high number of comparisons (2000 time points × 2 condition), to reduce the extent of type-1 errors, we randomly permuted the source waveforms for 3000 times and time points were considered as significant when at least 10 successive t-tests reached the significance threshold (*p* < 0.05; two-tailed).

To assess changes in ERPs related to the Go/NoGo task, we used ERP amplitude as dependent variable, taking into consideration only the non-TMS trials. This analysis was performed in the same way as for the TEPs. Namely, global analysis was performed with non-parametric, cluster-based permutation statistics conducted at each time point, for each individual electrode. This analysis was conducted by comparing ERP peak-to-peak amplitude within their five time windows (i.e., − 850 to − 750 ms for P1 related to the fixation point, FIX-P1; − 750 to − 600 ms for N1 related to the fixation point, FIX-N1; − 150 to − 100 ms for N1 related to the cue appearance, CUE-N1; − 100 to 0 ms for P1 related to the cue appearance, CUE-P1; 0–300 ms for N2 related to the cue, CUE-N2). Analysis at the local level was conducted over a cluster of medial fronto-central electrodes resulting significant from the cluster-based analysis (see results) and was performed with a 2-way repeated-measures ANOVA with “task condition” and “ERP peak” (FIX-P1, FIX-N1, CUE-N1, CUE-P1, CUE-N2) as within-subject factors. Post-hoc analysis were conducted with dependent t-test corrected with Sidak method.

We then assessed linear relations between task-related behavioural measures, i.e., RT (we excluded FA and MISS because of their very low occurrence, see results), and task-related neurophysiological measures, i.e., the modulation of SMA–M1 connectivity (NoGo–Go PLV difference) and M1 cortical activity (NoGo–Go TEP amplitude difference) during TMS perturbation. This analysis was conducted with Pearson’s or Spearman’s correlation coefficient, depending on the distribution of data. Finally, we were interested to assess whether the TMS-evoked N100 peak-to-peak amplitude, which we found to be a sensitive marker of inhibition (see results and discussion), was predicted by SMA–M1 connectivity. To this aim, we used a stepwise forward multiple regression using the TMS-evoked N100 amplitude as dependent variable, and the PLV values in the four frequencies (theta, alpha beta and gamma) in the four task phases (preparation, cue elaboration, TMS perturbation and task response) as predictors.

## Data Availability

The data of this study are available from the corresponding author upon reasonable request.
